# Impact of on-field repeated sprint training on aerobic fitness and anaerobic performance in football athletes: A matched-pair design

**DOI:** 10.1371/journal.pone.0323573

**Published:** 2025-06-09

**Authors:** Ramita Gupta, Moattar Raza Rizvi, Ankita Sharma, Shweta Sharma, Abdul Rahim Shaik, Mohamed K. Seyam, Shahid Raza, Mastour Saeed Alshahrani, Batool Abdulelah Alkhamis, Hani Hassan Alnakhli, Lama Moraya Alsalm, Fuzail Ahmad, Irshad Ahmad

**Affiliations:** 1 Department of Physiotherapy, School of Allied Health Sciences, Manav Rachna International Institute and Studies (MRIIRS), Faridabad, India; 2 College of Healthcare Professions (CoHP), D.I.T University, Dehradun, Uttarakhand, India; 3 Department of Physiotherapy, Amity Institute of Health Allied Sciences, Amity University, Noida, India; 4 Maharishi Markandeshwar Institute of Physiotherapy & Rehabilitation, Maharishi Markandeshwar University (MMU), Ambala, India; 5 Department of Physical Therapy & Health Rehabilitation, College of Applied Medical Sciences, Majmaah University, Almajmaah , Saudi Arabia; 6 Centre for Physiotherapy and Rehabilitation Sciences, Jamia Millia Islamia, New Delhi, India.; 7 Department of Medical Rehabilitation Sciences, College of Applied Medical Sciences, King Khalid University, Abha, Saudi Arabia; 8 Physical Therapy Department Saudi Germany Hospital, Abha, Saudi Arabia; 9 Respiratory Care Department, College of Applied Sciences, Almaarefa University, Riyadh, Saudi Arabia; Ordu University, TÜRKIYE

## Abstract

**Background:**

Repeated sprint ability (RSA) is essential for football performance, especially in maintaining high-intensity efforts throughout a match. Repeated sprint training (RST) improves both aerobic and anaerobic capacities; however, its effects on players in different positional roles remain underexplored, particularly with regard to tailored conditioning protocols.

**Objective:**

This study assessed the impact of on-field RST on physical performance metrics, including aerobic capacity (VO₂max), sprint speed (10m DASH), vertical jump height (VJH), and power output, with a focus on positional differences among forwards, defenders, and goalkeepers.

**Methods:**

Forty male football players (aged 18–25 years) were purposively sampled and matched by position before allocation into experimental (RST) and control groups. The experimental group completed a structured four-week RST program, while the control group continued routine football training involving technical, tactical, and endurance drills. Pre- and post-intervention assessments included the Cooper Test (aerobic capacity), VO₂max, VJH, power output, and 10m DASH.

**Results:**

After 4 weeks of RST, significant mprovements were observed in the experimental group. VO_2_max increased by 4.4 ml/kg/min (95% CI: 2.9 to 6.0; p < 0.001, d = 1.31), and 10m sprint time decreased by 0.32 seconds (95% CI: -0.45 to -0.19; p < 0.001, d = 1.36) in forwards. VJH improved significantly (p < 0.001) among defenders (Δ = 3.44 cm, 95% CI: 1.76 to 5.12, d = 1.06), while power improvements were most notable in defenders (Δ** =** 43.44W, 95% CI: 28.62 to 58.26, d = 1.00). Goalkeepers showed modest, non-significant improvements. Significant positional differences were identified for VJH and power output (p < 0.001).

**Conclusion:**

RST significantly enhanced physical performance metrics, particularly for forwards and defenders. The findings emphasize the importance of positional specificity in training programs to optimize football performance.

## Introduction

Football, or soccer as it is known in many parts of the world, stands as one of the most physically demanding and globally beloved sports. The game demands players to integrate speed, agility, strength, endurance, and tactical awareness, creating a symphony of physiological and cognitive skills on the field. During a standard 90-minute match, players typically cover 10–13 kilometers, alternating between high-intensity sprints, jogging, and walking [1]. The intermittent nature of football requires players to perform repeated explosive actions, often with minimal recovery time, testing both their aerobic and anaerobic energy systems [2].

Repeated sprint ability (RSA), the capacity to perform successive high-intensity sprints with short recovery periods while maintaining performance, is a key determinant of football success [3,4]. This ability distinguishes elite players by enabling sustained high-intensity efforts during critical match moments. Enhancing RSA is central to football-specific conditioning, with repeated sprint training (RST) emerging as a popular methodology in sports science. RST, characterized by short-duration, maximal-effort sprints interspersed with brief recovery intervals, effectively engages both anaerobic and aerobic energy systems [5].

The role of RST in improving football performance has been well-documented. Buchheit et al. (2010) highlighted that repeated shuttle sprint training enhanced the ability to change direction, while explosive strength training improved vertical jump performance in elite adolescent soccer players [5]. Similarly, Dello Iacono et al. (2015) observed increased change-of-direction speed and aerobic capacity among elite young soccer players after incorporating RST into their training regimes [6]. The benefits of RST are not limited to physiological metrics but extend to game-specific performance, allowing players to maintain higher levels of activity during matches [2]. Despite its effectiveness, implementing RST in football training presents challenges. Variations in work-to-rest ratios, sprint distances, and session durations can significantly influence training outcomes. For instance, Beato et al. (2019) compared straight sprint RST and change-of-direction RST, observing better improvements in 10-meter sprint and agility performance with the latter [7]. However, the optimal design of RST protocols remains a subject of ongoing research, particularly for field-based applications requiring minimal equipment [8].

Football positions such as forward, defender, and goalkeeper are associated with distinctly different physical profiles, tactical roles, and energy system demands [9]. Forwards typically engage in frequent high-intensity sprints; defenders alternate between sustained efforts and quick accelerations; goalkeepers perform explosive, reactive movements. These differences suggest that players may respond differently to conditioning interventions such as repeated sprint training (RST). Despite this, many existing RST studies overlook positional distinctions, limiting their applicability to real-world training. To address this gap, our study stratified players by position and investigated whether RST elicits varying physiological adaptations across roles—an approach that directly informs the development of more individualized, performance-oriented training strategies.

Recent research has highlighted the physiological relevance of repeated sprint ability (RSA) in professional football, showing that it closely relates to neuromuscular performance and fatigue markers. RSA performance has been significantly associated with mechanical parameters such as countermovement jump height and post-exercise blood lactate accumulation, suggesting that both power output and metabolic stress are key contributors to repeated sprint performance [10]. Additionally, acceleration intensity during repeated sprint exercises has been shown to influence both internal and external training load demands, reinforcing the importance of individualized training prescriptions to optimize performance outcomes [11]. Observational studies in youth soccer matches have also demonstrated that players often perform repeated sprint sequences under fatigue, emphasizing the ecological validity of RSA as a performance marker [5]. These findings support the application of RSA protocols in football training, particularly when considering positional demands and fatigue management.

Repeated sprint training (RST) offers distinct advantages over other high-intensity modalities such as traditional HIIT and plyometrics. While HIIT targets aerobic-anaerobic systems over longer intervals, RST involves short, maximal sprints with brief recoveries, better replicating the demands of match play [5,12]. Plyometric training, on the other hand, enhances neuromuscular explosiveness and may serve as a complementary approach [13]. Effective RST requires structured periodization to prevent overtraining and ensure progressive gains [14]. Although most studies focus on male amateurs, adaptations are also observed in elite and female players, though these groups may benefit from tailored protocols due to physiological differences [7,15]. Thus, RST is broadly applicable when individualized to player profiles and training levels.

This study aimed to evaluate the effects of a structured four-week on-field repeated sprint training (RST) program on aerobic fitness and anaerobic performance among football players, with a particular focus on understanding positional differences. It was hypothesized that RST would significantly improve aerobic capacity (VO₂max), sprint speed (10m DASH), vertical jump height (VJH), and power output compared to routine football training, with forwards and defenders expected to demonstrate greater improvements than goalkeepers. This approach highlights the positional specificity of RST, laying the foundation for developing targeted training strategies that equip players with the tools to excel in their roles. Unlike laboratory-based studies, this research emphasizes simplicity and accessibility, offering a practical and cost-effective solution that enables coaches to implement effective training protocols without relying on specialized equipment. Football’s dynamic and competitive nature necessitates such innovative methodologies, and this study aspires to contribute to the growing body of knowledge on RST by providing evidence-based recommendations to enhance performance. By addressing the gaps in current research and offering practical implementation strategies, this work seeks to empower coaches and players to maximize their potential on the field.

## Methods

### Study design

This study employed a quasi-experimental matched-pair design. Football players were purposively sampled based on playing positions (forwards, defenders, and goalkeepers) and matched for age and BMI. Allocation into experimental and control groups ensured positional representation. The study adhered to the Declaration of Helsinki principles and was approved by the Department Ethics Committee at Faculty of Allied Health Sciences, Manav Rachna International Institute of Research and Studies (Ethical approval number: MRIIRS/FAHS/PT/2022–23/S-16). The protocol was registered with the Clinical Trials Registry of India (CTRI/2023/05/052749). Written informed consent was obtained from all participants after they were fully briefed on the study’s objectives, procedures, and potential risks. Participants were assured of their confidentiality and right to withdraw from the study at any stage without providing a reason.

### Sample size calculation

The sample size calculated using G*Power 3.1.9.4 software [16], applying a matched-pair (dependent samples) t-test design suitable for pre- and post- intervention comparison within groups. Based on previous RST intervention studies [17] and assuming a medium effect size (dz = 0.5), a total sample of 34 participants was estimated to achieve 80% power at an alpha level of 0.05. To account for potential attrition, 10% dropout rate was considered to get the final sample size of 40 participants, ensuring sufficient statistical power even with participant attrition.

### Participants recruitment

**Fig 1:**
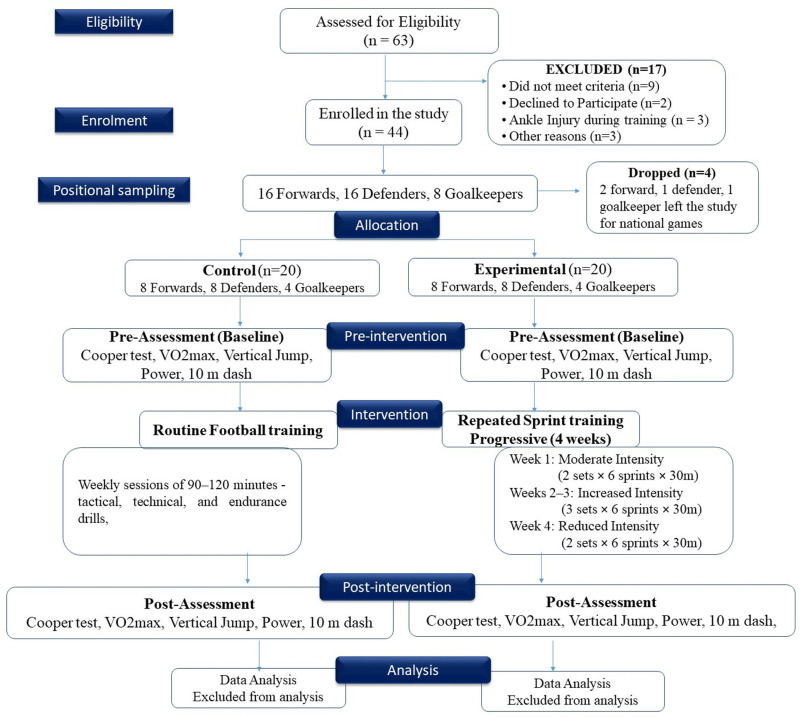
Flow chart of the study design.

Male football players aged 18–25 years were recruited between January 10 and June 10, 2023, using purposive sampling to ensure representation of different playing positions, including forwards, defenders, and goalkeepers, from district-level football teams and academies. Eligibility criteria included a minimum of two years of playing experience, a training frequency of 4–5 days per week, and no use of performance-enhancing supplements. Players with prior surgeries requiring more than six months of bed rest or orthopedic injuries involving lower limb joints within the past six months were excluded. A total of 44 players were initially enrolled, of whom 40 completed the full study protocol and were included in the final analysis (Fig 1). The final sample consisted of 16 forwards, 16 defenders, and 8 goalkeepers, with equal positional distribution across control and experimental groups (8 forwards, 8 defenders, and 4 goalkeepers per group).

Participants were recruited through direct outreach to local football teams and academies, leveraging existing networks and player databases. Initial screening ensured that players met all inclusion and exclusion criteria. After eligibility confirmation, participants were fully briefed on the study objectives, protocol, and potential risks, and written informed consent was obtained. Following enrolment, players were assigned to experimental and control groups using a matched-pair approach, based on age, BMI, and playing position, to ensure positional and physiological equivalence at baseline. Due to the nature of the intervention, participant blinding was not feasible; however, outcome assessors were blinded to group allocation to minimize measurement bias.

### Interventions

The experimental group participated in a structured four-week on-field Repeated Sprint Training (RST) program aimed at enhancing aerobic endurance, anaerobic power, and sprint speed. Training sessions were conducted twice weekly, beginning with a 15-minute warm-up involving dynamic stretches and mobility drills. The RST protocol was progressive: in Week 1, players completed two sets of 6 × 30-meter sprints; in Weeks 2 and 3, the training intensity increased to three sets of 6 × 30-meter sprints; and in Week 4, the volume was reduced to two sets of 6 × 30-meter sprints to minimize neuromuscular fatigue. Each sprint was followed by a 20-second recovery period, and a five-minute rest was provided between sets to simulate match-play demands and to stress both phosphagen and glycolytic energy systems [18]. The choice of a four-week training duration was based on prior research showing that short-term RST interventions conducted twice weekly over four weeks can induce significant improvements in intermittent running performance, aerobic capacity, and sprint speed among team-sport athletes [14,19]. These studies demonstrated that such timeframes are sufficient to elicit measurable physiological adaptations without overtraining or compromising player availability during competitive phases.

The control group continued their routine football training, which typically included weekly sessions lasting 90–120 minutes. These sessions involved a 15–20 minute warm-up (light jogging, dynamic stretching, and mobility drills), technical drills (ball control, passing, dribbling, and shooting), tactical training (team strategies, formations, and set-piece practice), physical conditioning (endurance runs, agility drills, and strength exercises), and small-sided games (~20–30 minutes) to simulate match scenarios. Both groups engaged in identical tactical and technical football drills to ensure consistency in training load, with the only difference being the progressive RST component introduced for the experimental group.

### Data collection and testing procedure

Data collection and testing were conducted at two time points: baseline (pre-intervention) and post-intervention, after four weeks of training. Standardized protocols were employed to ensure consistency and reliability across all assessments. To reduce measurement errors, all testing procedures were piloted before actual implementation, and all researchers were trained to ensure inter-rater reliability. The same researcher administered pre- and post-tests for each participant to minimize variability. Participants were thoroughly briefed on the testing procedures before each session to enhance compliance and understanding. All tests were performed in controlled conditions, minimizing external variability, with trained researchers closely monitoring each participant to ensure safety and adherence to the protocol.

**Aerobic endurance** was assessed using the Cooper test [20]. To reduce measurement variability, the test was performed on a flat, standardized 400-meter outdoor track under consistent environmental conditions. Participants were familiarized with the procedure in advance to minimize learning effects. The total distance was recorded in meters using the calibrated distance markers, and the same trained observer conducted both the pre- and post-intervention assessments to ensure consistency. Participants ran continuously for 12 minutes, aiming to cover the maximum possible distance. VO_2_ max was estimated using the formula: VO_2_ max = (Distance covered in meters - 504.9) ÷ 44.73 [21]. Group-based testing was used to simulate competitive conditions and enhance motivation.

**Speed** was evaluated using the 10-meter sprint test [22]. To minimize measurement error, the test was conducted on a flat, non-slip surface with clearly cones placed exactly 10 meters apart. Participants were familiarized with the procedure prior to testing to reduce learning effects. Each trial began from a standardized standing start position, and timing was recorded using the same calibrated stopwatch and trained observer throughout both pre- and post-intervention sessions to ensure consistency. Participants performed three trials with adequate rest between attempts to prevent fatigue-induced variability. The fastest time was recorded as the final score. Instructions emphasized proper sprinting techniques and verbal encouragement was provide to ensure maximum effort across all trials.

**Anaerobic power** was measured using the vertical jump test [23]. To minimize measurement error, the test was conducted indoors on a level surface with consistent lighting and flooring. Each participant stood flat-footed against a wall-mounted measuring tape to ensure fixed reference points across all trials. The standing reach height was recorded by having the participant extend their dominant arm upward with fingertips fully extended. Participants then performed a maximal jump from a semi-squat position, reaching as high as possible against the wall. The difference between the standing reach height and the maximum jump height was recorded as the vertical jump height. Each participant completed three trials, with the best performance used for analysis. Power output was calculated using the Lewis formula: Power (W) = √(4.9 × Weight in kilograms × Vertical Jump Height in meters × 9.81) [24]. This test provided insights into lower limb power and explosive capabilities.

All testing sessions were conducted in a professional sports facility equipped with the necessary tools, including cones, stopwatches, tape measures, and markers. Researchers ensured precise and unbiased measurements by adhering to rigorous protocols. Participants were carefully monitored to prevent injuries and to ensure they adhered to the testing protocols. Data were meticulously recorded during both baseline and post-intervention phases to facilitate accurate analysis. This comprehensive and structured approach to data collection ensured the reliability and validity of the results, enabling a robust evaluation of the effects of repeated sprint training on football players’ physical performance.

### Statistical analysis

Statistical analyses were conducted using SPSS software (version 25.0, IBM Corp., Armonk, NY, USA). Descriptive statistics (mean ± standard deviation) were calculated for all outcome variables across different positions (forward, defender, goalkeeper) and groups (control vs. experimental). Prior to inferential analyses, the assumption of normality was assessed using the Shapiro–Wilk test for all outcome measures at pre- and post-intervention time points. All variables met the assumption of normality, justifying the use of parametric tests. Independent t-tests were performed to compare baseline differences between the control and experimental groups, and paired t-tests were employed to evaluate pre-to-post changes within each group. A mixed-model repeated-measures ANOVA was utilized to examine the main effects of group and time, as well as their interaction (group × time). Additionally, the influence of position and group × position interactions were analyzed using multivariate analysis. Post-hoc Bonferroni pairwise comparisons were applied to significant effects to further explore positional differences. Effect sizes were calculated using partial eta-squared (η²) for multivariate analyses and Cohen’s d for paired comparisons. The effect sizes for η² were categorized as small (0.01–0.05), medium (0.06–0.13), and large (≥0.14), while Cohen’s d was interpreted as small (0.2–0.49), medium (0.5–0.79), and large (≥0.8). Statistical significance was set at p < 0.05.

## Results

The matched-pair allocation ensured no significant differences in age and BMI between the control and experimental groups across all positions (Table 1). Among forwards, the control group had a mean age of 21.86 ± 3.24 years, while the experimental group averaged 21.5 ± 2.62 years (t = 0.24, p = 0.82). For defenders, the mean age was 21.86 ± 2.54 years in the control group and 22.88 ± 2.42 years in the experimental group (t = -0.79, p = 0.44). Goalkeepers in the control group had a mean age of 22.75 ± 2.87 years, compared to 22.5 ± 2.65 years in the experimental group (t = 0.13, p = 0.90). For forwards, the control group had a mean BMI of 22.91 ± 2.85, while the experimental group had a BMI of 22.44 ± 2.61 (t = 0.33, p = 0.74). Among defenders, the control group had a mean BMI of 23.27 ± 1.52 compared to 22.65 ± 1.19 in the experimental group (t = 0.88, p = 0.40, d = 0.45). For goalkeepers, the control group’s BMI was 23.53 ± 1.11, while the experimental group had a BMI of 22.91 ± 3.69 (t = 0.33, p = 0.76, d = 0.23). These results indicate baseline equivalence between groups in age and BMI.

**Table 1:** Baseline Comparison of Outcome Variables between Control and Experimental Groups across Positions Using Independent t-Test.

Outcome Variable	Position	Time	Control Mean ± SD	Experimental Mean ± SD	t	p	MD
**Age (Years)**	Forward	Pre	21.86 ± 3.24	21.50 ± 2.62	0.24	0.82	0.36
	Defender		21.86 ± 2.54	22.88 ± 2.42	-0.79	0.44	-1.02
	Goalkeeper		22.75 ± 2.87	22.5 ± 2.65	0.13	0.90	0.25
**BMI (kg/m**^**2**^)	Forward	Pre	22.91 ± 2.85	22.44 ± 2.61	0.33	0.74	0.47
	Defender		23.27 ± 1.52	22.65 ± 1.19	0.88	0.40	0.61
	Goalkeeper		23.53 ± 1.11	22.91 ± 3.69	0.33	0.76	0.63
**Cooper Test (m)**	Forward	Pre	2591.71 ± 392.48	2611.63 ± 173.25	-0.13	0.9	-19.91
		Post	2593.86 ± 389.09	2810.25 ± 133.68	-1.48	0.16	-216.39
	Defender	Pre	2594.71 ± 362.54	2556.5 ± 221.84	0.25	0.81	38.21
		Post	2596.43 ± 366.95	2721 ± 183.03	-0.85	0.41	-124.57
	Goalkeeper	Pre	2487.5 ± 265.75	2590 ± 311.23	-0.5	0.63	-102.5
		Post	2511.75 ± 228.08	2787.75 ± 185.28	-1.88	0.11	-276
**VO_2_ max (ml/kg/min)**	Forward	Pre	46.65 ± 8.78	47.1 ± 3.87	-0.13	0.9	-0.45
		Post	46.7 ± 8.7	51.54 ± 2.99	-1.48	0.16	-4.84
	Defender	Pre	46.72 ± 8.11	45.87 ± 4.96	0.25	0.81	0.86
		Post	46.76 ± 8.2	49.54 ± 4.09	-0.85	0.41	-2.79
	Goalkeeper	Pre	44.33 ± 5.94	46.62 ± 6.96	-0.5	0.63	-2.29
		Post	44.86 ± 5.1	51.03 ± 4.14	-1.88	0.11	-6.17
**VJH (cm)**	Forward	Pre	26.83 ± 3.62	30.45 ± 4.14	-1.79	0.1	-3.62
		Post	27.08 ± 3.91	32.2 ± 4.72	-2.26	0.04	-5.11
	Defender	Pre	34.4 ± 8.38	31.59 ± 7.23	0.7	0.5	2.81
		Post	34.44 ± 8.36	35.04 ± 7.41	-0.15	0.89	-0.59
	Goalkeeper	Pre	43.86 ± 5.17	36.31 ± 8.7	1.49	0.19	7.55
		Post	43.95 ± 5.15	38.45 ± 11	0.91	0.4	5.5
**Power (Watts)**	Forward	Pre	761.45 ± 71.07	827.01 ± 121.77	-1.25	0.23	-65.55
		Post	764.67 ± 69.66	850.44 ± 130.1	-1.56	0.14	-85.77
	Defender	Pre	853.11 ± 90.92	818.22 ± 92.08	0.74	0.47	34.9
		Post	853.69 ± 90.75	861.66 ± 75.27	-0.19	0.86	-7.97
	Goalkeeper	Pre	989.05 ± 50.84	970.52 ± 114.6	0.3	0.78	18.53
		Post	990.07 ± 51.54	996 ± 139.63	-0.08	0.94	-5.93
**10m DASH (s)**	Forward	Pre	2.09 ± 0.41	2.37 ± 0.41	-1.28	0.22	-0.27
		Post	2.13 ± 0.37	2.04 ± 0.34	0.44	0.67	0.08
	Defender	Pre	2.11 ± 0.4	2.08 ± 0.28	0.14	0.89	0.02
		Post	2.12 ± 0.38	1.87 ± 0.28	1.49	0.16	0.25
	Goalkeeper	Pre	2.73 ± 0.29	2.16 ± 0.44	2.15	0.07	0.56
		Post	2.71 ± 0.29	1.97 ± 0.46	2.73	0.03	0.74

Independent t-tests revealed no significant differences between the experimental and control groups at baseline across all outcome measures, confirming initial group equivalence (Table 1). Specifically, p-values were as follows: Cooper Test (p = 0.90 for forwards, p = 0.81 for defenders, p = 0.63 for goalkeepers), VO₂max (p = 0.90 for forwards, p = 0.81 for defenders, p = 0.63 for goalkeepers), vertical jump height (p = 0.10 for forwards, p = 0.50 for defenders, p = 0.19 for goalkeepers), power output (p = 0.23 for forwards, p = 0.47 for defenders, p = 0.78 for goalkeepers), and 10m DASH (p = 0.22 for forwards, p = 0.89 for defenders, p = 0.07 for goalkeepers). Post-intervention comparisons similarly showed no statistically significant between-group differences in most cases, although performance gains were consistently observed within the experimental group. Post-test p-values were as follows: Cooper Test (p = 0.16 for forwards, p = 0.41 for defenders, p = 0.11 for goalkeepers), VO₂max (p = 0.16 for forwards, p = 0.41 for defenders, p = 0.11 for goalkeepers), vertical jump height (p = 0.04 for forwards, p = 0.89 for defenders, p = 0.40 for goalkeepers), power output (p = 0.14 for forwards, p = 0.86 for defenders, p = 0.94 for goalkeepers), and 10m DASH (p = 0.67 for forwards, p = 0.16 for defenders, p = 0.03 for goalkeepers). These findings suggest that while within-group changes were significant in the experimental group, between-group comparisons were less conclusive, possibly due to limited sample size within each positional subgroup (Table 1).

### Cooper test (Meters)

The experimental group demonstrated significant improvements in distance covered following repeated sprint training (RST) for forwards (ΔM = 198.62, p < 0.001, d = 1.31), defenders (ΔM = 164.50, p < 0.001, d = 1.01), and goalkeepers (ΔM = 197.75, p = 0.14, d = 0.53) indicating large aerobic gains for field players and moderate, though statistically non-significant, gains for goalkeepers. While the improvement in goalkeepers did not reach statistical significance, the effect size suggests potentially meaningful clinical benefit, especially given the smaller subgroup sample. In contrast, the control group exhibited negligible changes across all positions (Table 2). Mixed-model repeated-measures ANOVA revealed a significant main effect of the group (p = 0.03) confirming that RST elicited superior aerobic adaptations overall, although no significant position or group × position interaction effects were found (Table 3). Post-hoc comparisons (Table 4) showed no significant differences in Cooper Test improvements between positions, including forwards vs. defenders (ΔM = 46.40, p = 0.954), forwards vs. goalkeepers (ΔM = 59.52, p = 0.944), and defenders vs. goalkeepers (mean difference = 13.12, p = 0.999) potentially due to limited statistical power and risk of type I error associated with multiple comparisons. These findings highlight that although RST uniformly benefits aerobic performance, the extent of improvement may not significantly differ by position within the study’s sample constraints.

**Table 2:** Paired t-Test Results for Pre-to-Post Intervention Differences in Outcome Variables across Positions.

Outcome Variable	Position	Group	Pre (Mean ± SD)	Post (Mean ± SD)	Mean Difference	t	p	d
**Cooper Test (m)**	Forward	Control	2591.71 ± 392.48	2593.86 ± 389.09	2.14	0.32	0.75	0.03
		Experimental	2611.63 ± 173.25	2810.25 ± 133.68	198.62	5.80	<0.001	1.31
	Defender	Control	2594.71 ± 362.54	2596.43 ± 366.95	1.71	0.16	0.87	0.02
		Experimental	2556.50 ± 221.84	2721.00 ± 183.03	164.50	4.43	<0.001	1.01
	Goalkeeper	Control	2487.50 ± 265.75	2511.75 ± 228.08	24.25	0.97	0.40	0.19
		Experimental	2590.00 ± 311.23	2787.75 ± 185.28	197.75	1.93	0.14	0.53
**VO_2_max (ml/kg/min)**	Forward	Control	46.65 ± 8.78	46.70 ± 8.70	0.04	0.32	0.75	0.03
		Experimental	47.10 ± 3.87	51.54 ± 2.99	4.44	5.80	<0.001	1.31
	Defender	Control	46.72 ± 8.11	46.76 ± 8.20	0.03	0.16	0.87	0.02
		Experimental	45.87 ± 4.96	49.54 ± 4.09	3.67	4.43	<0.001	1.01
	Goalkeeper	Control	44.33 ± 5.94	44.86 ± 5.10	0.54	0.97	0.40	0.19
		Experimental	46.62 ± 6.96	51.03 ± 4.14	4.42	1.93	0.14	0.53
**VJH (cm)**	Forward	Control	26.83 ± 3.62	27.08 ± 3.91	0.25	0.82	0.43	0.10
		Experimental	30.45 ± 4.14	32.20 ± 4.72	1.74	6.47	<0.001	1.46
	Defender	Control	34.40 ± 8.38	34.44 ± 8.36	0.04	0.37	0.72	0.05
		Experimental	31.59 ± 7.23	35.04 ± 7.41	3.44	4.47	<0.001	1.06
	Goalkeeper	Control	43.86 ± 5.17	43.95 ± 5.15	0.08	2.03	0.13	0.41
		Experimental	36.31 ± 8.70	38.45 ± 11.00	2.13	1.54	0.22	0.44
**Power (Watts)**	Forward	Control	761.45 ± 71.07	764.67 ± 69.66	3.21	0.79	0.45	0.09
		Experimental	827.01 ± 121.77	850.44 ± 130.10	23.43	6.07	<0.001	1.37
	Defender	Control	853.11 ± 90.92	853.69 ± 90.75	0.57	0.39	0.71	0.06
		Experimental	818.22 ± 92.08	861.66 ± 75.27	43.44	4.38	<0.001	1.00
	Goalkeeper	Control	989.05 ± 50.84	990.07 ± 51.54	1.01	1.83	0.16	0.38
		Experimental	970.52 ± 114.60	996.00 ± 139.63	25.47	1.55	0.21	0.45
**10m DASH (s)**	Forward	Control	2.09 ± 0.41	2.13 ± 0.37	0.03	1.48	0.18	0.17
		Experimental	2.37 ± 0.41	2.04 ± 0.34	-0.32	-6.05	<0.001	1.36
	Defender	Control	2.11 ± 0.40	2.12 ± 0.38	0.01	1.01	0.34	0.12
		Experimental	2.08 ± 0.28	1.87 ± 0.28	-0.21	-4.63	<0.001	1.07
	Goalkeeper	Control	2.73 ± 0.29	2.71 ± 0.29	-0.01	-0.40	0.71	0.05
		Experimental	2.16 ± 0.44	1.97 ± 0.46	-0.19	-7.65	<0.001	1.72

**Table 3:** Multivariate Analysis of Variance (MANOVA) Examining Group, Position, and Group × Position Interaction Effects on Outcome Variables.

Outcome	Time	Group Effect			Position Effect			Group × Position Interaction		
		F	p	η²	F	p	η²	F	p	η²
Cooper Test (m)	Pre	0.08	0.78	0.002	0.12	0.89	0.01	0.15	0.86	0.01
	Post	5.04	0.03	0.14	0.14	0.87	0.01	0.23	0.80	0.01
VO₂max (ml/kg/min)	Pre	0.08	0.78	0.002	0.12	0.89	0.01	0.15	0.86	0.01
	Post	5.04	0.03	0.14	0.14	0.87	0.01	0.23	0.80	0.01
Vertical Jump (cm)	Pre	1.08	0.31	0.03	8.47	<0.001	0.35	2.19	0.13	0.12
	Post	0.00	0.98	0.00	7.56	<0.001	0.32	1.59	0.22	0.09
Power (Watts)	Pre	0.02	0.90	0.00	10.16	<0.001	0.39	1.14	0.33	0.07
	Post	1.01	0.32	0.03	9.56	<0.001	0.37	0.74	0.49	0.04
10m DASH (s)	Pre	0.69	0.41	0.02	2.23	0.12	0.12	3.21	0.05	0.17
	Post	9.10	<0.001	0.22	2.60	0.09	0.14	2.36	0.11	0.13

**Table 4:** Post-Hoc Comparisons of Positional Effects on Outcome Variables across Groups Using Multivariate Analysis.

Outcome Variable	Comparison	Mean Difference	SE	p	95% CI
Cooper Test (m)	Forward vs Defender	46.40	98.63	0.954	-202.08 to 294.88
	Forward vs Goalkeeper	59.52	118.26	0.944	-238.40 to 357.44
	Defender vs Goalkeeper	13.12	118.26	0.999	-284.80 to 311.04
VO₂max (ml/kg/min)	Forward vs Defender	1.04	2.21	0.954	-4.52 to 6.59
	Forward vs Goalkeeper	1.33	2.64	0.944	-5.33 to 7.99
	Defender vs Goalkeeper	0.29	2.64	0.999	-6.37 to 6.95
Vertical Jump (cm)	Forward vs Defender	-4.14	2.32	0.231	-9.99 to 1.71
	Forward vs Goalkeeper	-11.33	2.78	0.001	-18.34 to -4.32
	Defender vs Goalkeeper	-7.19	2.78	0.043	-14.20 to -0.18
Power (Watts)	Forward vs Defender	-38.09	34.77	0.629	-125.69 to 49.51
	Forward vs Goalkeeper	-183.37	41.69	<0.001	-288.40 to -78.34
	Defender vs Goalkeeper	-145.28	41.69	0.004	-250.31 to -40.25
10m DASH (s)	Forward vs Defender	0.10	0.13	0.834	-0.23 to 0.42
	Forward vs Goalkeeper	-0.26	0.15	0.274	-0.65 to 0.13
	Defender vs Goalkeeper	-0.36	0.15	0.078	-0.75 to 0.03

### VO₂max (ml/kg/min)

Post-training, VO₂max significantly increased in the experimental group for forwards (ΔM = 4.44, p < 0.001, d = 1.31), defenders (ΔM = 3.67, p < 0.001, d = 1.01), and goalkeepers (ΔM = 4.42, p = 0.14, d = 0.53), while the control group showed minimal changes (Table 2). Although the improvement in goalkeepers did not reach statistical significance, the moderate effect size suggests a potentially meaningful physiological adaptation that may be underpowered due to the smaller sample. Mixed-model repeated-measures ANOVA identified a significant main effect of the group (p = 0.03) but no significant position or group × position interaction effects (Table 3), indicating that VO₂max improvements were consistent across roles. Post-hoc analysis (Table 4) indicated no significant positional differences, with forwards vs. defenders (ΔM = 1.04, p = 0.954) and forwards vs. goalkeepers (ΔM = 1.33, p = 0.944) which may reflect both the physiological generalizability of RST across positions and a potential risk of type I error due to multiple comparisons

### Vertical jump height (cm)

Significant post-training improvements in VJH were observed in the experimental group for forwards (ΔM = 1.74, p < 0.001, d = 1.46) and defenders (ΔM = 3.44, p < 0.001, d = 1.06), suggesting large and clinically meaningful improvements in explosive lower-limb power. In contrast, goalkeepers exhibited modest, non-significant improvements in vertical jump height (ΔM = 2.13, p = 0.22, d = 0.44), reflecting the limited transferability of RST to the specific demands of their position, which rely more on reactive agility and lateral quickness than on repeated sprinting (Table 2). Mixed-model repeated-measures ANOVA revealed significant positional effects both pre- (p < 0.001) and post-training (p < 0.001) but no significant group or group × position interaction effects (Table 3). Post-hoc comparisons (Table 4) showed forwards had significantly lower VJH than goalkeepers (ΔM = -11.33, p = 0.001) and defenders had significantly lower jumps than goalkeepers (ΔM = -7.19, p = 0.043), while forwards and defenders showed no significant differences (ΔM = -4.14, p = 0.231). These findings emphasize the positional specificity of neuromuscular adaptations and highlight that while RST enhances vertical power in field players, its utility may be limited for goalkeepers

### Power output (Watts)

The experimental group exhibited significant improvements in power for forwards (ΔM = 23.43, p < 0.001, d = 1.37) and defenders (ΔM = 43.44, p < 0.001, d = 1.00), while goalkeepers showed no significant changes (ΔM = 25.47, p = 0.21, d = 0.45), suggesting that RST was more effective in enhancing explosive lower-limb power in outfield players compared to goalkeepers. Mixed-model repeated-measures ANOVA showed significant positional effects both pre- (p < 0.001) and post-training (p < 0.001) indicating inherent role-based differences in power output, however, no significant group or group × position interaction effects. Post-hoc analysis identified significant differences in power between forwards and goalkeepers (ΔM = -183.37, p < 0.001) and defenders and goalkeepers (ΔM = -145.28, p = 0.004), with no significant differences between forwards and defenders (ΔM = -38.09, p = 0.629). These findings underscore the positional specificity of power demands in football and suggest that while statistical significance was achieved in outfield positions, the moderate effect in goalkeepers may still reflect clinically meaningful adaptation.

### 10m DASH (Seconds)

Sprint speed significantly improved in the experimental group, with reductions in 10m DASH time for forwards (ΔM = -0.32, p < 0.001, d = 1.36), defenders (ΔM = -0.21, p < 0.001, d = 1.07), and goalkeepers (ΔM = -0.19, p < 0.001, d = 1.72). The control group exhibited minimal changes. Mixed-model repeated-measures ANOVA revealed a significant main effect of the group (p < 0.001) and a significant group × position interaction effect (p = 0.05), though the position effect itself was not statistically significant. Post-hoc comparisons showed no significant differences in 10m DASH improvements between forwards and defenders (ΔM = 0.10, p = 0.834) or forwards and goalkeepers (ΔM = -0.26, p = 0.274), while differences between defenders and goalkeepers approached significance (ΔM = -0.36, p = 0.078). These results suggest that RST may elicit meaningful improvements in sprint speed across all positions, including goalkeepers, despite positional roles differing in their sprinting demands. The large effect size in goalkeepers implies potential clinical relevance, even where statistical significance between groups was not fully established.

## Discussion

This study highlights the effectiveness of a four-week repeated sprint training (RST) program in improving in aerobic endurance, anaerobic power, sprint performance, among football players with the experimental group showing greater improvements than the control group. While statistically significant changes were observed, the inclusion of effect sizes (Cohen’s d) allowed for evaluation of clinical relevance, highlighting meaningful improvements particularly in forwards and defenders. These findings align with and extend current understandings of RST as a potent training modality for athletes engaged in high-intensity, intermittent sports like football, where repeated high-effort activities are coupled with brief recovery periods. However, the adaptations among goalkeepers were less pronounced, suggesting that RST protocols may need to be tailored to better address position-specific demands such as reaction time, lateral agility, and explosive movement patterns.

RST is a specialized form of high-intensity interval training (HIIT) involving short, maximal-effort sprints followed by brief recovery intervals [25]. Its relevance in team sports like football stems from its resemblance to match demands, where players perform repeated sprints, accelerations, and high-intensity efforts with intermittent recovery [14,26]. RST effectively mimics these demands, activating both aerobic and anaerobic energy systems [5,27]. Mechanistically, it enhances mitochondrial biogenesis, oxidative enzyme activity, and capillary density, improving oxygen delivery and utilization [28]. It also improves glycolytic and phosphocreatine energy system efficiency, enabling repeated explosive efforts with reduced fatigue [29]. Neuromuscularly, RST promotes better motor unit recruitment, synchronization, and firing rates, boosting explosive strength and sprint capacity [30]. These adaptations make RST a versatile tool for improving high-intensity performance in football.

The findings revealed significant improvements in the Cooper Test performance, indicating enhanced aerobic endurance in the experimental group, particularly among forwards and defenders. These outcomes suggest that RST effectively improves oxygen uptake and utilization [31] by inducing cardiovascular adaptations such as increased stroke volume, cardiac output, and capillary density [32,33]. The repeated high-intensity bouts in RST sessions also promote better lactate clearance and buffering capacity [28,34] enabling athletes to sustain higher intensities during dynamic match conditions.

The increase in VO₂max observed in the experimental group underscores the cardiovascular benefits of RST reflecting improved oxygen transport and metabolic efficiency during high-intensity activities [15,35]. These enhancements are attributed to physiological adaptations such as improved phosphocreatine resynthesis, mitochondrial function and lactate clearance [5,36]. These mechanism enabling sustained performance and efficient recovery, particularly benefiting forwards and defenders who face greater aerobic and anaerobic demands [37]. Despite these improvements, post-hoc analysis revealed no significant positional differences, suggesting that RST provides universal aerobic and anaerobic benefits across all playing roles.

Significant improvements in vertical jump height (VJH) in the experimental group, highlighting the effectiveness of RST in enhancing anaerobic power. As a proxy for explosive strength and neuromuscular efficiency—critical for actions like heading, jumping, and tackling – VJH improvements suggest enhanced recruitment of fast-twitch muscle fibers and motor unit synchronization [13,35,38,39]. These neuromuscular adaptations underscore RST’s role in developing explosive performance capacities in football. Similarly, the power output increased notably in the experimental group especially among forwards and defenders, reflecting improved anaerobic performance. Power is essential for explosive football actions such as accelerations, direction changes, and shooting or tackling [12]. These improvements results from enhanced glycolytic enzyme activity and muscle contractility stimulated by the high-intensity RST [27,40], enabling players to generate greater force during key match moments.

Sprint performance, as measured by the 10m DASH, significantly improved in the experimental group, particularly among forwards and defenders. The reduction in sprint times reflects enhanced neuromuscular coordination, stride efficiency, and improved phosphocreatine resynthesis and lactate clearance, enabling repeated high intensity efforts with reduced fatigue [41]. Such adaptation are critical in football where speed and acceleration can influence match outcomes [42]. Additionally, RST appears to enhance sprint repeatability contributing to both performance and endurance under fatigue conditions [43].

The results of this study underscore positional differences in adaptations to RST. Forwards and defenders demonstrated the most substantial improvements across aerobic and anaerobic measures, likely due to their greater physical demands during matches. Forwards rely on explosive power and speed for attacking, while defenders require endurance and quick acceleration for maintaining defensive structures [3]. In contrast, goalkeepers exhibited modest improvements particularly in VJH and power output likely reflecting their position-specific demands, when emphasize agility and reaction time over sustained high-intensity efforts [40]. These findings highlights the need for tailoring RST to match the distinct physiological requirements of each playing role.

The performance improvements observed in this study can be attributed to combination of aerobic, anaerobic, and neuromuscular adaptations. Aerobic enhancements such as increased cardiac output, stroke volume and mitochondrial density improve oxygen delivery and utilization during sustained high-intensity activity [44]. Anaerobic adaptations such as improved phosphocreatine resynthesis increased glycolytic capacity and enhanced muscle contractility support repeated explosive efforts [45]. Additionally, neuromuscular adaptations like improved motor unit recruitment and synchronization enhance sprint performance and power output. Together, these mechanism reinforce RSTs value as a well-rounded conditioning method for football players.

## Practical implications and applications

The results of this study offer actionable insights for football coaches and trainers. The matched-pair design confirmed RST’s effectiveness in improving key performance metrics, reinforcing its utility even without randomization. Positional representation ensured relevance to real-world team compositions. Forwards and defenders could incorporate sprint intervals with varying intensities and recovery periods, while goalkeepers may require additional agility-focused drills to complement RST. Coaches and trainers can integrate RST into pre-season and in-season training regimens to enhance players’ overall fitness and address position-specific demands. Furthermore, combining RST with structured strength training (e.g., resistance-based lower limb workouts) and aerobic conditioning can amplify gains in power and endurance. For forwards and defenders, who benefit most from RST, incorporating sprint intervals with varied intensities and recovery periods can optimize performance outcomes. For goalkeepers, RST may need to be supplemented with agility and reaction-based drills to address the unique demands of their position. To avoid overtraining and optimize recovery, it is recommended that fatigue management strategies be implemented, including proper rest intervals, load monitoring, and scheduled deloading weeks. These ensure neuromuscular recovery and sustained adaptations across the season.

Additionally, the short duration of the RST program in this study underscores its efficiency as a training intervention. In just four weeks, significant improvements were observed across multiple performance metrics, demonstrating that RST can yield meaningful results even within a limited timeframe. This makes it a valuable tool for teams with tight training schedules or limited preparation periods.

## Limitations and future directions

While this study provides valuable insights into the effects of RST on football performance, several limitations should be acknowledged. The relatively small sample size (n = 40), particularly within positional subgroups, limits the statistical power to detect subtle differences. Although assumptions for normality and homogeneity of variance were tested and met, the small sample size may still limit the robustness of parametric analyses such as ANOVA. Furthermore, the potential for false positives due to multiple comparisons cannot be entirely ruled out, even with post-hoc corrections, and should be considered when interpreting subgroup-level differences. Future studies should consider the use of non-parametric alternatives to account for potential deviations and improve statistical validity in small-sample contexts. The use of a purposive sampling strategy without randomization may introduce selection bias, which, combined with the inclusion of male-only participants, restricts the generalizability of the findings to broader populations, including female athletes or teams with different training regimens. Additionally, the study duration of four weeks was relatively short, potentially limiting the understanding of the long-term effects of RST. While statistically significant improvements were observed in several parameters, the effect size estimates (e.g., Cohen’s d) also highlight the clinical importance of these findings, particularly in sprint and aerobic performance. However, goalkeeper-specific adaptations, such as lateral movement and reaction time, were not directly assessed, and future research should incorporate performance measures tailored to the unique demands of this position. Future studies could address these limitations by employing larger, more diverse samples, randomization to minimize bias, and extending the intervention duration to capture sustained adaptations. Investigating the impact of RST on other physiological and performance outcomes, such as muscle fatigue, recovery dynamics, and psychological parameters, could further enhance the evidence base. Finally, combining RST with complementary conditioning protocols, such as strength training or agility-focused drills, may provide a more comprehensive understanding of how to optimize football performance across various skill levels and positional roles.

## Conclusion

This study demonstrates that on-field running-based repeated sprint training (RST) significantly enhances key physical performance metrics in football players, including aerobic capacity, anaerobic power, sprint speed, and vertical jump height. The results highlight the effectiveness of RST in improving the overall fitness of football players, demonstrating the value of a matched-pair design to ensure fairness and positional balance in non-randomized studies. Forwards and defenders experienced greater improvements compared to goalkeepers, suggesting that position-specific training may be beneficial for optimizing performance. The findings support the implementation of RST as an essential component of football conditioning programs, providing a simple and effective approach to improving player performance without requiring specialized equipment.
